# Potential Anti-COVID-19 Therapeutics that Block the Early Stage of the Viral Life Cycle: Structures, Mechanisms, and Clinical Trials

**DOI:** 10.3390/ijms21155224

**Published:** 2020-07-23

**Authors:** Rami A. Al-Horani, Srabani Kar, Kholoud F. Aliter

**Affiliations:** 1 Division of Basic Pharmaceutical Sciences, College of Pharmacy, Xavier University of Louisiana, New Orleans, LA 70125, USA; skar@xula.edu; 2 Department of Chemistry, School of STEM, Dillard University, New Orleans, LA 70122, USA; kal-horani@dillard.edu

**Keywords:** coronavirus, COVID-19, SARS-CoV-2, viral entry, viral fusion, ACE2, spike protein, TMPRSS2, endocytosis

## Abstract

The ongoing pandemic of coronavirus disease-2019 (COVID-19) is being caused by severe acute respiratory syndrome coronavirus-2 (SARS-CoV-2). The disease continues to present significant challenges to the health care systems around the world. This is primarily because of the lack of vaccines to protect against the infection and the lack of highly effective therapeutics to prevent and/or treat the illness. Nevertheless, researchers have swiftly responded to the pandemic by advancing old and new potential therapeutics into clinical trials. In this review, we summarize potential anti-COVID-19 therapeutics that block the early stage of the viral life cycle. The review presents the structures, mechanisms, and reported results of clinical trials of potential therapeutics that have been listed in clinicaltrials.gov. Given the fact that some of these therapeutics are multi-acting molecules, other relevant mechanisms will also be described. The reviewed therapeutics include small molecules and macromolecules of sulfated polysaccharides, polypeptides, and monoclonal antibodies. The potential therapeutics target viral and/or host proteins or processes that facilitate the early stage of the viral infection. Frequent targets are the viral spike protein, the host angiotensin converting enzyme 2, the host transmembrane protease serine 2, and clathrin-mediated endocytosis process. Overall, the review aims at presenting update-to-date details, so as to enhance awareness of potential therapeutics, and thus, to catalyze their appropriate use in combating the pandemic.

## 1. Introduction

### 1.1. The Pandemic’s Status and Its Clinical Presentations

At the end of 2019, the Chinese health authorities reported an outbreak of viral pneumonia cases of unidentified cause in Wuhan, Hubei, to the World Health Organization (WHO). After about a month, the WHO declared the outbreak as a public health emergency of international concern [1,2]. The outbreak was then declared by the WHO as a global pandemic on March 11, 2020 [3]. The outbreak has been caused by severe acute respiratory syndrome coronavirus-2 (SARS-CoV-2) and now it is widely known as the coronavirus disease-2019 (COVID-19). As of July 1 of this year, the virus has reportedly infected more than 10.9 million individuals worldwide with more than half a million human lives have been lost to the infection and/or its clinical complications [4].

SARS-CoV-2 is an enveloped, non-segmented, positive sense, and single stranded RNA virus which, upon infecting the respiratory system, causes pneumonia and acute respiratory distress syndrome (ARDS). The most common symptoms at the infection onset are cough, fatigue, myalgia, and fever [5,6]. Importantly, the disease is also associated with several extra-pulmonary symptoms. Gastrointestinal symptoms appear to be common and were reported in about 40% of patients in some studies [7,8]. Neurological symptoms have also been reported [9]. A group of COVID-19 patients also reported taste or olfactory disturbances [10], along with skin and eye symptoms [11,12].

Generally, it appears that about 80% of confirmed cases only experience mild illness, while the rest may suffer from severe pneumonia that requires hospital admission [13]. Although they initially appear stable, several reports suggested that patients who require hospital admission may rapidly deteriorate with severe hypoxia, leading to severe ARDS, which further requires admission to an intensive care unit (ICU) and mechanical ventilation [14]. These patients may also suffer from severe myocardial injury [15]. The progression of illness in these patients has been attributed to an excessive systemic inflammatory response and to an overactivation of the coagulation process, and the crosstalk between the two phenomena. On one hand, the excessive release of proinflammatory markers such as IL-1β, IL-6, IP-10, G-CSF, MCPI-1, MIP-1α, and TNF-α may eventually lead to multiorgan failure and death [2,16,17,18]. On the other hand, the hypercoagulable state of these patients, as indicated by the elevated levels of D-dimer and fibrinogen and the prolonged prothrombin time [19], also appears to be associated with poor clinical outcomes [20,21,22,23,24]. Reported thrombotic complications include intravascular disseminated coagulopathy, pulmonary embolism, and stroke [25,26,27,28].

Unfortunately, there are currently neither approved vaccines to protect individuals against the infection nor highly effective approved therapeutics to treat it. Yet, knowledge pertaining to the virus’s life cycle and the infection’s pathogenesis is rapidly evolving. Accordingly, researchers have swiftly responded to the pandemic by advancing potential therapeutics into clinical trials. While the treatment of mild COVID-19 cases requires no therapeutics or only supportive standard care, the treatment of more severe conditions necessitates the use of antiviral agents. Critically ill patients may also need anti-inflammatory and/or anticoagulant therapeutics. In this review, we describe the chemical structures and the mechanisms of action of potential antiviral therapeutics that block the early stage of the viral life cycle. We only include those therapeutics that are listed in clinicatrials.gov. They include both old drugs and new molecular entities and they are either small molecules or macromolecules. Some of these therapeutics possess activities beyond the inhibition of the early events of the viral life cycle.

### 1.2. The Life Cycle of SARS-COV-2 and Potential Drug Targets

**Figure 1:**
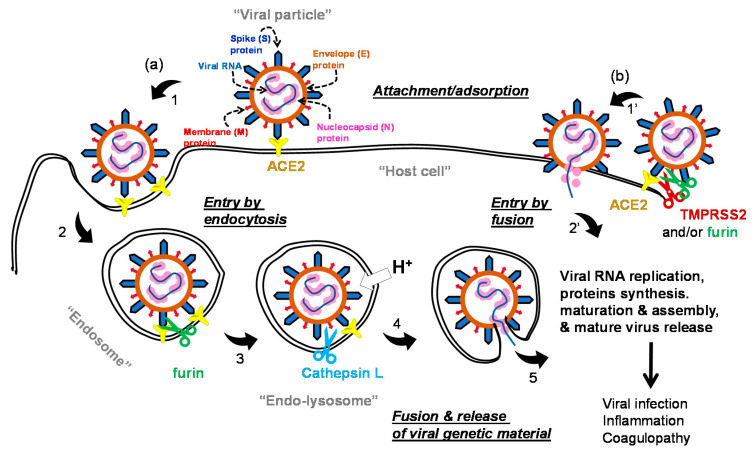
A representation of the viral particle of SARS-CoV-2 depicting the structural proteins: spike (S) protein, envelope (E) protein, membrane (M) protein, and nucleocapsid (N) protein. It also shows the early stage of the life cycle of the virus which starts by the spike S protein binding to the host cell ACE2, which is followed by viral entry. The viral entry takes place either by (**a**) endocytosis or (**b**) direct fusion. In endocytosis-mediated entry, the spike S protein activation appears to take place in endosomes by the action of furin. Further proteolysis and subsequent fusion occur because of the action of cathepsin L in endo-lysosomes. In direct fusion entry, the process is mediated by TMPRSS2 and/or furin; however, other trypsin-like proteases may also contribute. Either way, the RNA genetic material of the virus is released, and the late stage of the life cycle subsequently takes place by RNA replication, viral protein synthesis, maturation, assembly, and release of the new virus.

The life cycle of the virus can generally be divided into early stage events and advanced stage events. In the early stage events (Figure 1), SARS-CoV-2 utilizes its spike (S) protein to bind to the host cell membrane via the membrane-embedded glycoprotein angiotensin converting enzyme 2 (ACE2) [2,29]. ACE2 is broadly expressed in vascular endothelium, respiratory epithelium, alveolar monocytes, and macrophages [30]. Other tissues may also express this receptor, such as the ileum, heart, kidney, and bladder [31]. Importantly, the spike S protein has two subunits: the S1 subunit which carries the receptor binding domain and the S2 subunit which has to be exposed for subsequent events [32,33]. Other coronaviruses can exploit other receptors, such as dipeptidyl peptidase-4 (DPP-4) or aminopeptidase N (APN) [34,35]; nevertheless, the literature is yet to report on alternative receptors for SARS-CoV-2. Following binding to ACE2, the virus enters the host cell by clathrin-mediated endocytosis process [36]. The bound virus spike S protein is then proteolytically activated by the action of furin so as to expose the S2 subunit [37]. Further processing appears to be promoted by the action of cathepsin L in (endo)lysosomes to eventually promote the viral envelope fusion with the host membrane and the release of the viral RNA [38]. The virus can also gain access to the host cell environment following the spike S protein-ACE2 complex activation by transmembrane protease serine 2 (TMPRSS2) which subsequently permits the viral fusion with the host cell and then the release of the viral genome [39]. Other proteases such as furin, trypsin, plasmin, factor Xa, and human airway trypsin-like protease may allegedly contribute to this process [40,41,42].

The RNA genetic material of SARS-CoV-2 has ~29,811 nucleotides which encode for about 29 proteins: structural proteins (four proteins), nonstructural proteins (NSPs; 16 proteins), and accessory proteins (nine proteins) [43,44]. The virus has four structural proteins of the envelope (E), membrane (M) proteins which form the viral envelope, nucleocapsid (N) protein which binds to the virus’s RNA genome, and the spike S protein which binds to human ACE2 as its host cell receptor. The NSPs domain is typically expressed as two polypeptides, which, upon processing, produce the NSPs, including papain-like protease (PL^pro^) (NSP3), main protease (M^pro^) (also known as 3-chymotrypsin-like protease (3CL^pro^); NSP5) [45], and RNA-dependent RNA polymerase (RdRp; NSP12) [46]. Initial processing of the two polypeptides is mediated by intracellular proteases, and then further propagated by the action of the viral PL^pro^ and M^pro^. The viral RdRp is also responsible for the replication of the viral genome and the amplification of the infection. Importantly, the viral RNA and the N protein are biosynthesized in the host cell cytoplasm, whereas other viral structural proteins (E, M, and S) are biosynthesized in the endoplasmic reticulum and then transported to the Golgi apparatus. The viral RNA–N complex and E, M, and S proteins are subsequently assembled in the endoplasmic reticulum–Golgi intermediate compartment, and then, form a mature virion via a budding process. The mature viral particle gets then released from the host cells via exocytosis [47,48,49].

Accordingly, the aim of therapeutics under development is to impede/block one or more events in the life cycle of the virus in order to hinder the propagation of the infection within the host cells. In fact, any of the described proteins or processes in the viral life cycle can be a target for drug discovery, design, and development efforts. In this review, we describe the molecules (Table 1) that are currently being tested in clinical trials to block the early events of the viral life cycle.

## 2. Potential Small Molecule Inhibitors of Early Viral Events in Clinical Trials

### 2.1. Quinoline-Based Drugs

**Figure 2:**
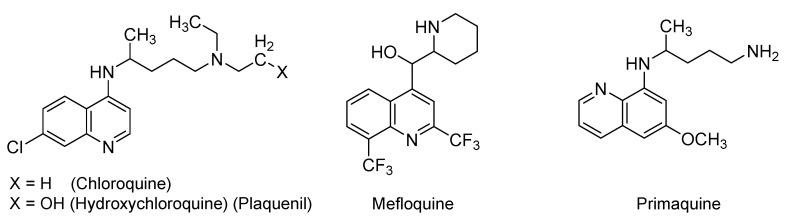
The chemical structures of quinoline-based antimalarial drugs that are being tested in COVID-19 patients.

A series of quinoline derivatives is being investigated in various settings for the prevention and/or treatment of COVID-19. Specific derivatives are depicted in Figure 2 and include chloroquine, hydroxychloroquine, mefloquine, and primaquine. In particular, chloroquine is a 4-aminoquinoline derivative that has been approved for oral use in the prevention and treatment of malaria and extraintestinal amebiasis. It has also been indicated for the treatment of lupus erythematosus. Hydroxychloroquine (Plaquenil, first approved by the U.S. FDA in 1955) is a more hydrophilic derivative of chloroquine which has also been orally used for malaria, lupus erythematosus, and rheumatoid arthritis [50,51]. Less common antimalarial drugs are mefloquine, which is 4-methanolquinoline derivative, and primaquine, which is 8-aminoquinoline derivative [52]. Chloroquine inhibits the heme (toxic product, ferriprotoporphyrin IX) conversion to hemazoin (nontoxic byproduct) in malarial trophozoites. Accumulation of the toxic heme kills the parasite. Furthermore, chloroquine can also diffuse into the parasite acidic vesicles and increases internal pH, leading to the inhibition of the parasite growth [50,51]. The antimalarial mechanisms of hydroxychloroquine and mefloquine are similar to that of chloroquine [53]. However, primaquine works differently by producing reactive oxygen species or by inhibiting the electron transport process in the parasite. Primaquine may also directly disrupt the protozoal DNA [54].

At the beginning of COVID-19 pandemic, the U.S. FDA issued an emergency use authorization for chloroquine and hydroxychloroquine [55]. The initial apparent efficacy of both drugs as antiviral agents has been documented in in vitro studies and attributed to a number of mechanisms. Specifically, a number of studies have revealed the in vitro activity of (hydroxy)chloroquine against SARS-CoV [56,57,58]. In fact, chloroquine was shown to potently block SARS-CoV-2 infection at a low-micromolar concentration (EC_50_ (half maximal effective concentration) = 1.13 μM; CC_50_ (half maximal cytotoxic concentration) > 100 μM) in VeroE6 cells [57]. Mechanistically, the in vitro studies showed that chloroquine affects SARS-CoV and other viruses, such as human immunodeficiency virus (HIV) and orthomyxoviruses, by inhibiting quinone reductase which is involved in the biosynthesis of sialic acid, an acidic sugar that is important for the host ACE2 glycosylation. Thus, chloroquine appears to inhibit the glycosylation of the host ACE2 receptor, and subsequently, to interfere with the virus binding to the host receptor [55,59]. Furthermore, given their basic chemical characteristics, chloroquine and hydroxychloroquine have also been reported to increase the endosomal/lysosomal pH, and thus, can potentially disrupt the early viral life cycle events of entry and fusion [56,59]. Moreover, chloroquine and hydroxychloroquine were also reported to prevent, in vitro, the transport of SARS-CoV-2 from early endosomes to endo-lysosomes, which is necessary for the viral genome’s release [57]. Of note, chloroquine and hydroxychloroquine are widely distributed to different tissues, including the eyes, leukocytes, liver, kidneys, heart, and lungs where retention is relatively long [60]. The two drugs are also associated with immunomodulatory activities that can potentially help in mitigating the impacts of the cytokine storms in patients with viral infections [56,58,61,62,63,64]. Likewise, mefloquine and primaquine have also been assumed to demonstrate the same in vitro effects, given their structural similarities to chloroquine and hydroxychloroquine.

As far as active, recruiting, or completed interventional clinical trials for COVID-19, chloroquine has been considered in more than 35 clinical trials across the world. Its analog, hydroxychloroquine, has also been considered in more than 112 trials either alone or in combination with azithromycin, nitazoxanide, bromhexine, dexamethasone, ivermectin, intravenous famotidine, or lopinavir/ritonavir. Some of these trials are testing its use as a treatment, while others are testing its use as a prophylactic. Furthermore, mefloquine is being evaluated in one interventional study (NCT04347031) alone or in combination with azithromycin or azithromycin and tocilizumab. Primaquine is being tried in one interventional study (NCT04349410) with hydroxychloroquine and clindamycin for COVID-19 pneumonia as a part of the Fleming Method for Tissue and Vascular Differentiation and Metabolism (FMTVDM) protocol.

Despite the initial claims about the potential efficacy of quinoline derivatives in COVID-19 patients, results pertaining to the use of chloroquine and hydroxychloroquine in these patients remain largely inconclusive. The majority of trials have involved patients with mild or moderate COVID-19, and limited trials have been reported in patients with severe and critical disease. For example, a small randomized, controlled trial (N = 22) in China compared the effect of receiving chloroquine (500 mg twice daily for 10 days) with receiving lopinavir/ritonavir (400 mg/100 mg twice daily for 10 days). Results indicated that chloroquine led to a faster RT-PCR conversion and recovery than lopinavir/ritonavir. Nevertheless, the study included few patients, and the median time from the symptoms’ onset to the treatment initiation was shorter in patients treated with chloroquine than in patients given lopinavir/ritonavir [65]. Furthermore, a double-blind, randomized phase 2b study in Brazil compared two different chloroquine regimens as adjunctive therapies for patients hospitalized (N = 81) with SARS-CoV-2 infection. Mortality by day 13 was found to be higher in the high-dose group (600 mg twice daily for 10 days; 39%) than in the low-dose group (450 mg twice daily for 1 day followed by 450 mg for four days; 15%). The study results suggested that the higher dosage of chloroquine should be avoided in critically ill COVID-19 patients because of its potential toxicity, particularly when it is concurrently administered with oseltamivir and azithromycin [66].

Furthermore, in another trial from China, hospitalized patients (N = 62) with mild CT-verified COVID-19 pneumonia were randomly assigned to the standard treatment group or to hydroxychloroquine (200 mg twice daily for five days) along with the standard treatment group. Relative to the control group, patients in the hydroxychloroquine group had a one-day-shorter mean duration of cough and fever. Of the control group, 13% suffered from progression of disease, whereas none of the hydroxychloroquine group experienced disease progression. About 81% of the drug-treated patients and approximately 55% of the control patients had moderate to significant improvement in the lung CT scans [67]. Along these lines, a multicenter, randomized, open-label study compared hydroxychloroquine (1200 mg/day for three days followed by hydroxychloroquine 800 mg/day for two weeks for patients (N = 148) with mild and moderate COVID-19 disease and three weeks for patients (N = 2) with severe COVID-19 disease) and the standard of care with the standard of care alone. The results indicated that hydroxychloroquine use in patients with mild to moderate COVID-19 does not provide additional therapeutic benefits [68].

An open-label, uncontrolled study of hydroxychloroquine with azithromycin was also recently reported in France [69]. Hospitalized COVID-19 patients (N = 11) were treated with hydroxychloroquine (600 mg/day for 10 days) and azithromycin (500 mg on day 1; then 250 mg/day on days 2–5). At time of treatment initiation, approximately 73% of patients had significant comorbidities, and about 91% of them had fever and received oxygen. Within five days, one patient died, two patients were transferred to the ICU, and the regimen was discontinued in one patient because of the adverse effect of QT interval prolongation. Nasopharyngeal samples continued to be PCR positive at days 5 and 6 in 80% of tested patients. Overall, this small study suggested that a regimen of hydroxychloroquine and azithromycin does not lead to a rapid viral clearance or provide any therapeutic benefit [69]. A very recent observational, retrospective cohort study analyzed data from patients (N = 368) hospitalized at the U.S. Veterans Health Administration medical centers with confirmed COVID-19. The study found no evidence that the use of hydroxychloroquine with or without azithromycin can reduce the risk of mechanical ventilation in these patients. An increase in the overall mortality rate was also reported in the hydroxychloroquine-only-treated patients [70]. Moreover, two retrospective studies analyzed clinical data for hospitalized COVID-19 patients ((N = 1438) [71] and (N = 1376) [72]) in New York so as to evaluate the effects of hydroxychloroquine treatment with or without azithromycin. Results suggested that, irrespective of azithromycin, hydroxychloroquine use does not decrease in-hospital mortality rate [71,72]. A large, randomized, controlled, adaptive trial has also evaluated the efficacies of six treatments for death prevention in hospitalized COVID-19 patients (N = 12,000) compared with the standard care alone (NCT04381936; RECOVERY). One arm included hydroxychloroquine (two 800 mg doses administered six hours apart followed by two 400 mg doses administered 12 and 24 h after the initial dose on day 1, and then 400 mg every 12 h for 9 days) (N = 1542 hydroxychloroquine-treated patients vs. N = 3132 patients control). The preliminary results have indicated that hydroxychloroquine does not provide significant benefits with respect to the 28-day mortality or the duration of hospitalization relative to the usual care alone [73,74]. Along these lines, a retrospective, observational cohort study also evaluated data for COVID-19 patients (N = 181) hospitalized with pneumonia at four French tertiary care centers over a 14-day period. The study revealed no statistically significant clinical benefits from using hydroxychloroquine within 48 h of hospitalization [75].

In a case series from France, hospitalized COVID-19 adults (N = 1061), described as asymptomatic or with upper/lower respiratory tract infections and treated with hydroxychloroquine (200 mg three times/day for 10 days), were evaluated against control individuals. The study found that nasopharyngeal PCRs were negative in 70% of the drug-treated patients, compared to only 12.5% of patients in the control group by the sixth day. Among the hydroxychloroquine-treated patients, about 57% of drug-treated patients and 100% of patients who were treated with hydroxychloroquine and azithromycin (500 mg on day 1 followed by 250 mg daily for the next four days) had negative nasopharyngeal PCRs by the sixth day. The study concluded that the hydroxychloroquine and azithromycin combination given prior to COVID-19 complications appears to be safe and associated with a low mortality rate [76]. Other clinical studies have reported similar inconclusive clinical outcomes when used in COVID-19 patients [77,78,79,80].

Prior to the use in COVID-19 patients, serious adverse effects have long been reported with the use of quinoline-based antimalarial drugs. Chloroquine has been linked to cardiac arrhythmias and retinopathy. Owing to its relatively increased hydrophilicity, hydroxychloroquine may be associated with less severe forms of the above adverse effects [81,82,83]. The use of mefloquine can lead to neuropsychiatric adverse reactions that may persist even after discontinuation [84]. Primaquine may cause hemolytic anemia in individuals deficient in glucose-6-phosphate dehydrogenase, and methemoglobinemia in NADH-methemoglobin reductase-deficient patients [85]. Importantly, the risk of cardiac arrhythmias continues to exist with the use of mefloquine and primaquine. In this direction, the risk of cardiac arrhythmias appears to be associated with the quinoline moiety that disrupts the function of certain cardiac ion channels, resulting in what is known as QT prolongation.

In the U.S., the NIH COVID-19 Treatment Guidelines Panel [86] and FDA [87] have issued multiple guidelines and safety alerts describing the use of chloroquine and hydroxychloroquine in COVID-19 patients and their potential adverse effects. In China, chloroquine has been suggested as a possible option and listed in the Chinese guidelines for COVID-19 treatment [88]. Nevertheless, the efficacy and safety of chloroquine and hydroxychloroquine (alone or in combination) for the treatment or the prevention of COVID-19 continue to be largely inconclusive [87,89]. In fact, effective 15 June 2020, the U.S. FDA has revoked the emergency use authorization that was issued on 28 March 2020 for hydroxychloroquine and chloroquine in COVID-19 patients. Considering the currently available scientific evidence, the FDA concluded that (1) chloroquine and hydroxychloroquine dosage regimens in the initial authorization are unlikely to produce the desired antiviral effect; (2) the initial results of decreasing the viral shedding with chloroquine or hydroxychloroquine have not been reliably reproduced; and (3) data from a large, randomized, controlled trial for hospitalized COVID-19 patients treated with hydroxychloroquine documented no evidence of clinical benefits with respect to the mortality rate, length of hospitalization, or need for mechanical ventilation [90].

### 2.2. Renin-Angiotensin-Aldosterone System (RAAS) Modifiers: Angiotensin Converting Enzyme Inhibitors (ACEIs) and Angiotensin Receptor Blockers (ARBs)

**Figure 3:**
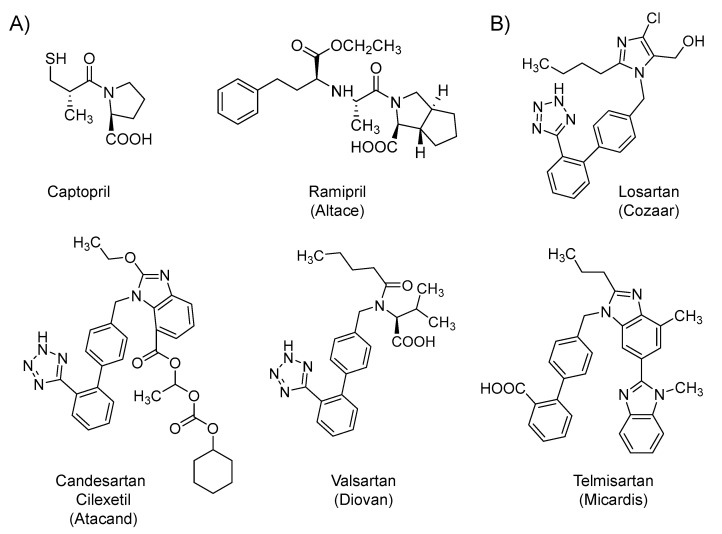
The chemical structures of RAAS modifiers. (**A**) The chemical structures of ACEIs and (**B**) The chemical structures of ARBs that are being tested with relevance to COVID-19.

Several RAAS modifiers are being considered in the context of COVID-19 patients. Captopril (1981) and ramipril (Altace; 1991) (Figure 3A) are synthetic small molecule inhibitors of angiotensin-converting enzyme, a Zn-containing proteolytic enzyme that is responsible for the biosynthesis of angiotensin II. Moreover, losartan (Cozaar; 1995), candesartan cilexetil (Atacand; 1998), valsartan (Diovan; 1996), and telmisartan (Micardis; 1998) (Figure 3B) are synthetic small molecule antagonists that competitively block angiotensin II receptors. The two classes directly and/or indirectly decrease the biological activity of angiotensin II, which is an octapeptide that, upon binding to its receptors, promotes vasoconstriction and increases the release of catecholamines and aldosterone hormone, leading to/or contributing to a series of cardiovascular diseases, including hypertension and heart failure. In other words, the drugs in the two classes limit the detrimental effects of angiotensin II on the cardiovascular system, and thus, they are commonly orally used in the management of hypertension and heart failure [91]. Currently, several clinical trials are underway to evaluate the effects of ACEIs (captopril and ramipril) and the ARBs (losartan, candesartan, valsartan, and telmisartan) in patients infected with COVID-19. Thus far, the use of drugs from the two classes appears to be controversial. Some scientists have suggested a hypothetical benefit, while others have cautioned due to the possibility of a harmful effect.

On the potential hypothetical benefit front, severe cases of COVID-19 are associated with refractory ARDS secondary to pneumonia. It is now known that the virus enters the respiratory airways and binds to its receptors, i.e., ACE2 protein on the membrane of the host cells by the means of the spike S protein [31,92,93]. The virus internalization appears to lead to a partial decrease or total loss of the enzymatic function of ACE2 in the alveolar cells. Because the ACE2 breaks down angiotensin II to angiotensin 1–7, the internalization of ACE2 results in excessive accumulation of the pro-inflammatory angiotensin II, which subsequently stimulates angiotensin II receptor, leading to increased pulmonary vascular permeability, the release of pro-inflammatory cytokines, and eventually severe lung pathology. Accordingly, ARBs have the potential to block the pathological consequences resulted from the accumulated angiotensin II. They block the excessive stimulation of angiotensin II receptor and they also upregulate ACE2, which will reduce angiotensin II concentration and increase the production of the protective vasodilator angiotensin 1–7. Therefore, these drugs have been proposed to prevent the development of ARDS and to decrease the risk of admission to ICU, the need for mechanical ventilation, and the overall mortality [92,93,94]. Furthermore, potential benefits may also arise because losartan can potentially block ACE2, and thus, may decrease the viral entry [95]; or because telmisartan can potentially inhibit the viral protein processing by inhibiting M^pro^, and thus, can inhibit the generation of mature and infective viral particles [95].

On the potential harm front, the harm may happen because the expression of ACE2 has been reported to increase in patients treated with ACEIs or ARBs [15,92,96]. Increased expression of ACE2 may potentially facilitate the viral entry during the early life cycle of COVID-19 [92,93]. However, a large observational study recently evaluated a cohort of hypertensive patients with COVID-19. The study concluded that the use of ACEIs or ARBs neither significantly increased the likelihood of COVID-19 incidence nor elevated its severity [97]. Another large, population-based case-control study concluded that treatment with RAAS modifiers did not significantly elevate the risk of COVID-19 or increase the infection severity [98]. Accordingly, the American Heart Association, American College of Cardiology, Heart Failure Society of America, and European Society of Cardiology have recommended continuing treatment with RAAS modifiers in patients who are being treated with them. Importantly, while the harmful effects appear to be unreal, the beneficial effects are yet to be proven.

### 2.3. Hydroxy Methylglutaryl Coenzyme A (HMG-CoA) Reductase Inhibitors: Atorvastatin and Simvastatin

**Figure 4:**
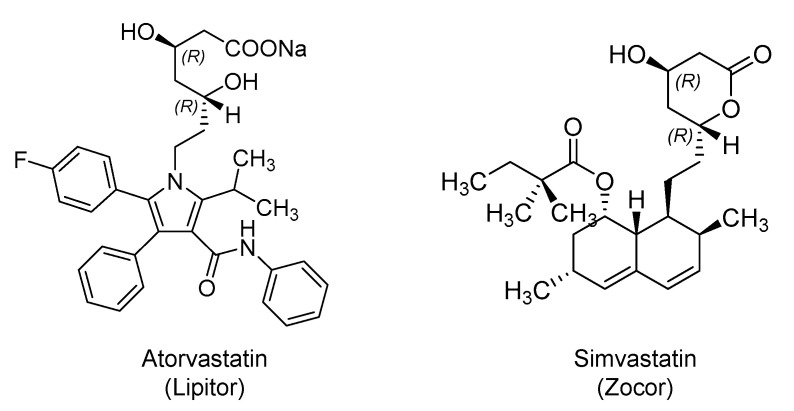
The chemical structures of HMG CoA reductase inhibitors being tested in clinical trials for potential therapeutic benefits in COVID-19 patients.

HMG-CoA reductase inhibitors, also known as statins, are orally bioavailable anti-hyperlipidemic drugs that block the hepatic biosynthesis of cholesterol by competitively inhibiting the enzyme that catalyzes the rate-limiting step. Their beneficial effects in patients infected with SARS-CoV-2 are yet to be proven. However, atorvastatin (Lipitor; 1996) and simvastatin (Zocor; 1991) (Figure 4) are being tried in few studies (atorvastatin; NCT04380402 and NCT04333407) and (simvastatin; NCT04343001 and NCT04348695) as potential therapies. Multiple rationales are presented in the literature to justify the statins’ potential benefits in COVID-19 patients.

It is well reported that SARS-CoV-2 enters the host cells via its interaction with ACE2 and the subsequent clathrin-mediated endocytosis. In earlier studies, statins were shown to reduce receptor-mediated endocytosis in opossum kidney cells [99] and in human kidney proximal tubular cells [100]. Thus, statins may block the entry of COVID-19 into human cells. Moreover, some studies suggested that statins may inhibit the M^pro^ of SARS-CoV-2 [101]. They have also been implicated in upregulating ACE2, which may potentially reduce the severity of ARDS, as reported previously [102]. Potential benefits in COVID-19 are also justified by statins’ anti-inflammatory and immunomodulatory effects that may prevent acute lung injury. Statins counteract endothelial dysfunction by modulating the ACE2/angiotensin-1–7/Mas receptor axis and angiopoietin/Tie-2 signaling axis [103]. Therefore, using statins as adjunct therapy has been proposed so as to accelerate the restoration of homeostasis, and thus, allow faster recovery [103]. Owing to the above effects, statins were also suggested to have beneficial effects in patients infected with Middle East respiratory syndrome-coronavirus (MERS-CoV) [104], Ebola virus [105], Zika virus [106], influenza [107], dengue virus [108], and others [109].

In this direction, a retrospective cohort study in Belgium assessed the effect of using statins in nursing home residents who were PCR-confirmed COVID-19 patients or suspected COVID-19 patients. The results indicated that statins use correlated with the absence of symptoms. About 45% of statins-treated patients were asymptomatic, whereas only 22% of those who did not receive statins were asymptomatic [110].

### 2.4. Bromhexine, Camostat, Nafamostat, and Clindamycin

**Figure 5:**
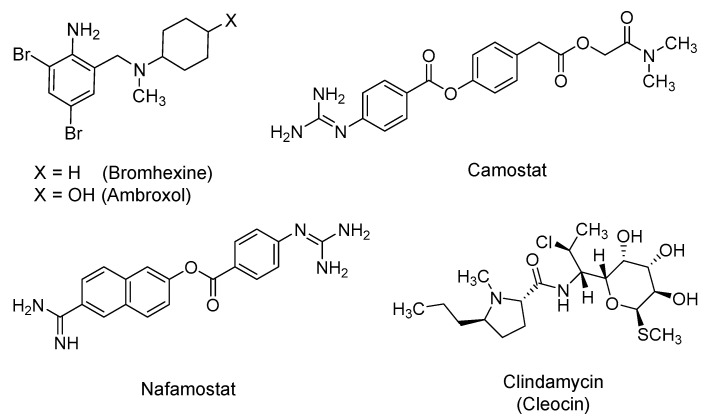
The chemical structures of TMPRSS2 inhibitors that are being tested in clinical trials for COVID-19 patients. The listed drugs potentially prevent the viral fusion with the host cell.

Bromhexine is a dibrominated phenylmethyl-cyclohexyl-amine derivative that is pharmaceutically prepared as a hydrochloride salt (Figure 5). It is an over-the-counter mucolytic agent that is reportedly used for pulmonary diseases with excessive mucous secretion and impaired mucous transport [111]. Bromhexine has been reported as an inhibitor of TMPRSS2 with an *IC_50_* (half maximal inhibitory concentration) value of 0.75 μM [112]. TMPRSS2 is an androgen regulated cell-surface, trypsin-like serine protease that is expressed in the human respiratory tract. It plays a critical role in the activation and invasion of the airway epithelium by influenza, SARS, and MERS viruses [113]. Likewise, SARS-CoV-2 cell entry has been found to be dependent on binding of the viral spike S protein to the host cell ACE2. The priming of the virus–host proteins complex by TMPRSS2 occurs at the S1/S2 Arg rich multi-basic site, following which the viral entry takes place. TMPRSS2 has also been implicated in regulating the viral assembly in the Golgi apparatus and the release of the mature virus from the host plasma membrane [40,114,115,116]. Thus, inhibiting TMPRSS2 by bromhexine is predicted to be a prophylactic strategy at the minimum. Furthermore, some of potential bromhexine’s beneficial effects can also be attributed to its 4-hydroxylated active metabolite that is known as ambroxol, owing to its ability to stimulate the synthesis and release of surfactants by type II pneumocytes. The secreted surfactants reduce the adhesion of mucus to the bronchial wall [117]. Currently, several clinical trials are underway to test orally administered bromhexine for the prevention and/or treatment of COVID-19 patients either alone (NCT04273763 and NCT04405999) or in combination with hydroxychloroquine (NCT04355026 and NCT04340349) or spironolactone (NCT04424134).

Camostat is another drug to be considered as a viral entry inhibitor. It is a guanidino-containing, nonspecific serine protease inhibitor (Figure 5). It was approved in Japan in 2006 for the treatment of esophagitis, pancreatitis, and cancer. It has also been found to be effective in treating fibrosis in kidney disease, liver disease, and pancreatitis [118,119,120]. Camostat was also shown to inhibit the replication of influenza virus [121]. Considering coronaviruses, camostat was found to be a TMPRSS2 inhibitor, and thus, partially blocked infection by SARS-CoV and human coronavirus NL63 in HeLa cell cultures [122]. Recently, it was also shown that camostat substantially decreases the infection of Calu-3 lung cells by SARS-CoV-2 [39]. In mice infected with SARS-CoV, camostat used at concentrations similar to the clinically used ones in humans diminished the mortality rate from 100% to 30–35% [123]. Following inhalation, camostat also, in vivo, attenuated airway epithelial sodium channel function by inhibiting a channel-activating protease. It also enhanced mucociliary clearance [124]. Accordingly, camostat, in the form of mesylate salt, is now being investigated in at least six clinical trials for COVID-19. In one of these studies, the drug is being tested in combination with hydroxychloroquine (NCT04355052). In addition, owing to its ability to inhibit trypsin-like proteases in the coagulation cascade, camostat is also being evaluated to treat COVID-19-associated coagulopathy (NCT04435015).

A third drug to be considered in this category is nafamostat, which is a guanidino and amidino-containing, nonspecific serine protease inhibitor (Figure 5). It is under investigation in two clinical trials (NCT04418128 and NCT04352400) to assess its efficacy in COVID-19 patients. In Japan, nafamostat has clinically been indicated for the treatment of pancreatitis, disseminated intravascular coagulation, and extracorporeal circulation anticoagulation [124,125,126,127]. Similar to camostat, nafamostat is an inhibitor of TMPRSS2, and thus, it was reported to block MERS-CoV S protein-facilitated viral membrane fusion with TMPRSS2-presenting lung Calu-3 host cells [128]. In cell culture experiments with simian Vero E6 cells, nafamostat inhibited SARS-CoV-2 infection at an *EC_50_* of 22.50 µM [57]. Most recently, nafamostat mesylate inhibited SARS-CoV-2 S-mediated entry into host cells with roughly 15-fold higher efficiency than camostat mesylate [129]. Importantly, in addition to pancreatic proteases, nafamostat also inhibits proteases in coagulation, fibrinolysis, the kallikrein–kinin system, and the complement cascade. Nafamostat has also been reported to inhibit the activation of protease-activated receptors [130]. Furthermore, nafamostat was also reported to inhibit lipopolysaccharide-induced nitric oxide production, apoptosis, IL-6, and IL-8 levels in cultured human trophoblasts [131]. It was also shown that nafamostat acts as an antioxidant against TNF-α-induced reactive oxygen species production [132]. Together, nafamostat possesses significant antiviral, anti-coagulation, anti-inflammatory, and antioxidant activities that may substantially benefit COVID-19 patients, as was reported recently in three cases [133].

Structural insights into the interactions of the three inhibitors described above with TMPRSS2 have been reported via computational study [134]. The molecular docking analysis revealed that camostat and nafamostat interact directly with residues His296, Asp435, and Ser441 in the catalytic domain of TMPRSS2. Bromhexine was found to interact with Gln438 residue and other residues in the active site of TMPRSS2 via hydrophobic interactions.

Along these lines, clindamycin (Cleocin; 1970) has also been identified via computational tools as a potential inhibitor of TMPRSS2, and thus, it may potentially interfere with the viral entry stage [95]. Clindamycin is a semisynthetic lincosamide antibiotic (Figure 5) with broad-spectrum antimicrobial activity. It reversibly binds to 50S ribosomal RNA subunits, preventing peptide bond formation, and thus inhibiting microbial protein synthesis. It was first approved in 1970 in the U.S. and is currently used topically and systemically as well as parenterally and orally. Clindamycin is mainly used to treat anaerobic infections, as in dental ones [135,136]. It is being evaluated in an interventional randomized trial known as the FMTVDM-directed COVID-19 treatment protocol (NCT04349410) in combination with hydroxychloroquine and primaquine. Other potential rationales for the use of clindamycin in COVID-19 patients can be attributed to its anti-inflammatory and immunomodulatory effects. Clindamycin was reported to modulate cytokine production in lipopolysaccharide-stimulated macrophages, to decrease TNF-α and IL-1β, to suppress the chemotaxis of polymorphonuclear leukocytes, to reduce inflammation, and to enhance the uptake of microorganisms by the host phagocytic cells [137]. It is also possible that it is being tested for antibacterial coverage in hospitalized patients with COVID-19-related pneumonia.

### 2.5. Umifenovir

**Figure 6:**
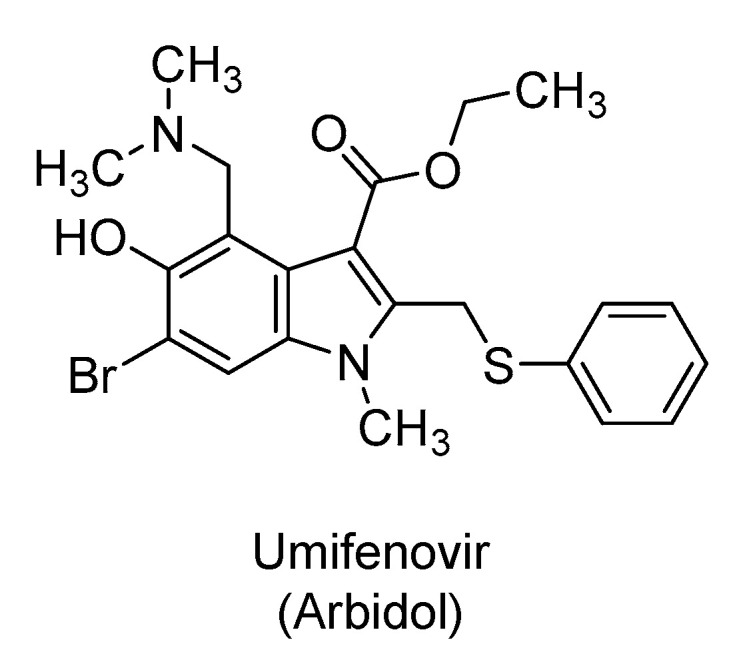
The chemical structure of umifenovir (abidol), an antiviral drug with multiple antiviral mechanisms of action. The drug is being tested alone or in combination with other potential therapeutics for the treatment of COVID-19 patients.

Umifenovir (Arbidol) is indole-3-ethylcarboxylate derivative (Figure 6) that is used in Russia, China, and other countries as a prophylactic or treatment for influenza and other respiratory infections [138,139]. It is a non-nucleoside antiviral agent with broad antiviral activity against non-enveloped and enveloped DNA and RNA viruses, including Ebola virus [140], Zika virus [141], hepatitis B and C viruses [142], and herpes simplex viruses [143], among others [144]. It is currently being evaluated in about five clinical trials for the treatment and/or prevention of SARS-CoV-2 infection. The drug is used pharmaceutically in the form of a hydrochloride salt.

Umifenovir is orally bioavailable hydrophobic drug that appears to target both the virus as well as the host components [145]. The drug is known as a dual-binding molecule because it can bind to both proteins and lipids, and thus, it can disrupt several macromolecular interactions affecting multiple stages of the viral life cycle [138]. In particular, the drug can form aromatic stacking interactions with aromatic amino acids of key viral glycoproteins important for recognition and fusion [146,147]. It can form similar interactions with the plasma membrane of the virus and that of the host which subsequently impacts viral entry, viral intracellular trafficking, and clathrin-mediated exocytosis [138,148,149]. Given its lipophilicity, the drug has been found to disrupt the assembly of lipoproteins deemed critical for hepatitis C virus assembly/maturation. It has also been proposed to disrupt a variety of lipid-protein interactions in the membranous web where the viruses of the *Flaviviridae* family replicate [138]. Recently, computational work suggested that umifenovir may interact with multiple SARS-CoV-2 proteins of NSP7/NSP8 complex, NSP14, NSP15, E-channel, and/or spike S protein [95]. Furthermore, structural and molecular dynamics studies have recently suggested that umifenovir binds to the SARS-CoV-2 spike S protein and prevents its trimerization, which is a key step for viral–host cell adhesion [150].

The use of umifenovir in clinical settings has been reported in a few studies. A retrospective cohort study in China in COVID-19 adults (N = 50) suggested a better viral inhibition with umifenovir (200 mg three times/day) compared to lopinavir/ritonavir (400 mg/100 mg twice a day for a week). The patients also received interferon α-2b. COVID-19 was undetectable in 50% of umifenovir-treated patients versus 23.5% of lopinavir/ritonavir-treated patients, at the seventh day after hospitalization. At the fourteenth day, the viral load was undetectable in all umifenovir-treated patients, yet it was undetectable in only 44% of patients treated with lopinavir/ritonavir. The virus RNA test was positive for a shorter period in patients treated with umifenovir [151]. Another retrospective cohort study (N = 33) suggested a more favorable outcome with the triple therapy of lopinavir/ritonavir/umifenovir relative to lopinavir/ritonavir [152]. At the seventh day time point, SARS-CoV-2 was undetectable in nasopharyngeal specimens in 75% of patients treated with the triple therapy versus only 35% of patients treated with lopinavir/ritonavir alone. At the same time, chest CT scans improved in 69% of patients treated with lopinavir/ritonavir/umifenovir, whereas only 29% of patients treated with lopinavir/ritonavir alone showed improved chest CT scans. At the fourteenth day, the virus was undetectable in 94% of patients treated with the triple therapy versus 53% of patients treated with lopinavir/ritonavir alone [152]. However, a third retrospective cohort study of non-ICU adult patients (N = 81) with COVID-19 in China reported no statistically significant difference in SARS-CoV-2 clearance between the umifenovir-treated patients and the control group [153]. Furthermore, an open-label, prospective, randomized, multicenter study involving COVID-19 patients (N = 240) in China compared favipiravir (1600 mg twice a day for the first day followed by 600 mg twice a day for nine days) with umifenovir (200 mg three times a day for 10 days). The study revealed that the recovery rate was higher in favipiravir-treated patients than in umifenovir-treated patients [154]. Moreover, a randomized, partially blinded, and single-center trial in China evaluated the efficacy of umifenovir with standard care versus lopinavir/ritonavir with standard care versus the standard care alone in hospitalized, mild/moderate COVID-19 adult patients (N = 86). The study revealed no statistically significant difference in the mean time for SARS-CoV-2 RNA positive-to-negative conversion in respiratory specimens and no statistically significant difference in therapeutic outcomes between patients treated with umifenovir or lopinavir/ritonavir and those who were treated with the standard care without antiviral therapy [155].

### 2.6. Macrolides: Azithromycin, Clarithromycin, and Carrimycin

**Figure 7:**
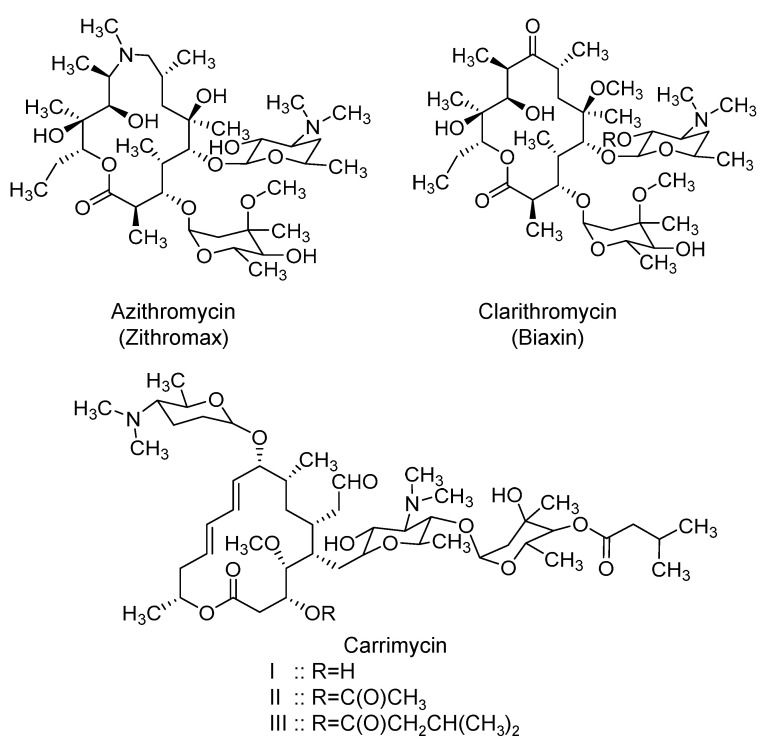
The chemical structures of three macrolides that are currently being evaluated in clinical trials for the treatment of COVID-19 patients. The macrolides also exhibit anti-inflammatory/ immunomodulatory effects.

Azithromycin (Zithromax) is a semi-synthetic broad-spectrum macrolide antibiotic (Figure 7) that was initially approved by the U.S. FDA in 1991. Mechanistically, it predominantly inhibits microbial protein synthesis by binding to the 50S subunit of microbial ribosomal RNA [156,157]. The drug is available in oral and parenteral dosage forms. Relative to other macrolides, azithromycin has a long elimination half-life of up to 72 h in adults and extensive tissue distribution with volume of distribution of ~32 L/kg. Azithromycin distributes well into skin, lungs, the sputum, tonsils, and the cervix [156,157]. Azithromycin has been used in chronic respiratory inflammatory diseases owing to its immunomodulatory effects, in addition to its antimicrobial effects. Potential major adverse effects include hearing loss and cardiac arrhythmia [158]. The drug is currently being evaluated across the whole world in about 85 interventional clinical trials for the prevention and/or treatment of COVID-19 pneumonia. It is being tried alone and in combination with hydroxychloroquine (most frequent), amoxicillin/clavulanate, atovaquone, mefloquine, or tocilizumab.

The rationale for its use in SARS-CoV-2 patients can be attributed to its antiviral and anti-inflammatory activities. The activity of azithromycin was in vitro tested against SARS-CoV-2. Azithromycin inhibited the viral replication at a concentration range of 5–10 µM, and it worked synergistically with hydroxychloroquine. Effective concentrations of azithromycin were comparable to those of hydroxychloroquine [159]. Another in vitro study found that azithromycin inhibited the viral replication with an *EC_50_* value of 2.12 µM, and its *CC_50_* value was >40 µM [160]. Importantly, the molecular mechanism of azithromycin in SARS-CoV-2 has also been studied. Azithromycin (100 µM) has been found to inhibit the cleavage of the spike protein, and thus, has prevented the viral entry in IB3-1 CF cells. This has been attributed to azithromycin’s ability to significantly reduce the level/activity of furin, an enzyme that cleaves the spike protein [161]. Furthermore, it has been found that treatment of CF bronchial epithelial cells with azithromycin (1–100 µM) increased the pH of the trans-Golgi network and that of the recycling endosome; both arguably are important for viral protein packaging, replication, and spread. The increased pH of the trans-Golgi network was also suspected to alter the glycosylation of ACE2. The modified pattern of ACE2 glycosylation may inhibit SARS-CoV-2 from binding to the host cells [161]. Beyond SARS-CoV-2, azithromycin has been reported to be active against Zika [162,163,164], Ebola [165], and influenza H1N1 viruses [166,167]. Azithromycin has also been reported to reduce inflammation markers production and to increase the macrophages’ phagocytosis ability [168,169]. Macrolide antibiotics are reported to diminish the production of pro-inflammatory cytokines such as IL-6 and TNF-α [170]. It has also been documented that the CF cells treatment with azithromycin (1–100 µM) reduces the basal levels of IL-8 secretion [161].

Importantly, azithromycin has been used for several lung diseases, including asthma, chronic obstructive pulmonary disease, bronchiectasis, interstitial lung diseases, [171,172], idiopathic pulmonary fibrosis [173,174], diffuse pan-bronchiolitis [175], and ARDS [176]. It has also been used to provide antibacterial protection in hospitalized patients with COVID-19 pneumonia in Wuhan, China [14]. In France, azithromycin has been used in combination with hydroxychloroquine in an open-label nonrandomized study (N = 36) [177], an open-label uncontrolled study (N = 11) [69], an uncontrolled observational study (N = 80) [79], and a large uncontrolled observational study (N = 1061) [76]. Thus far, the results reported are inadequate to evaluate the potential therapeutic benefits of azithromycin in COVID-19 patients. As indicated before, there are more clinical trials being performed to evaluate the efficacy of azithromycin for a wide spectrum of COVID-19 patients.

Likewise, clarithromycin, another macrolide (Figure 7), has been reported to help COVID-19 patients in combination with hydroxychloroquine [178]. Its potential is currently being tested in an open-label, non-randomized clinical trial (ACHIEVE; NCT04398004).

Another macrolide under investigation is carrimycin. Structurally, it appears to be a mixture of derivatives of spiramycin, a macrolide antibiotic similar to azithromycin. A survey of peer-reviewed literature and patent literature suggests that the 4”-OH group in spiramycin is modified to yield isovaleryl spiramycin I, II, and III (Figure 7) which together account for 60% of the whole mixture. Mechanistically, it inhibits the microbial protein synthesis by targeting the ribosomal RNA components [179,180]. Carrimycin (also known as biotechspiramycin or shengjimycin) has been developed by a synthetic biology technology and has shown potent antibacterial activity and significant inhibitory activity against mycoplasma and chlamydia. It also appears to be effective against drug-resistant Gram-positive bacteria, some Gram-negative bacteria, *Candida albicans*, and *Mycobacterium tuberculosis*, among others. It potentially has immunomodulatory action and antiproliferative activity [179,180]. Carrimycin was approved by the Chinese National Medical Products Administration in 2019 to treat upper respiratory infection. It is being tested in a multicenter, randomized, open-controlled study in China to determine its efficacy and safety in patients with COVID-19 (NCT04286503). The justifications for its use are similar to those described for azithromycin.

### 2.7. Ivermectin

**Figure 8:**
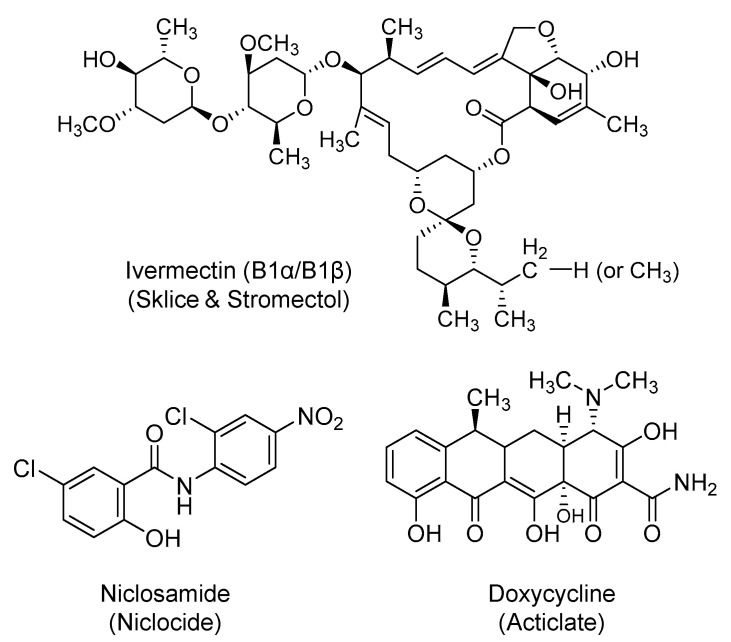
The chemical structures of a broad-spectrum antiparasitic agent (ivermectin), an aryl nitro-based antiparasitic agent (niclosamide), and a broad-spectrum antibacterial agent (doxycycline). Doxycycline also exhibits potential anti-inflammatory effects.

It is a semisynthetic, macrocyclic lactone (Figure 8) with broad-spectrum activity against various parasites. Ivermectin is used topically and systemically for a host of parasitic diseases, including onchocerciasis, strongyloidiasis, headlice, and rosacea, among others. It is an *O*-glycoside that was first approved in the U.S. in 1996 and is being marketed under the names of Sklice and Stromectol. It binds selectively and with high affinity to the glutamate-gated chloride ion channels in invertebrate muscle and nerve cells, leading to enhanced permeability to chloride ions, hyperpolarization, paralysis, and death of the parasite [181,182]. Currently, orally administered ivermectin is being studied alone or in combination with nitazoxanide or hydroxychloroquine for the treatment of COVID-19 patients in about 25 clinical trials.

Ivermectin has demonstrated a broad in vitro antiviral activity against both RNA and DNA viruses [183,184]. Previously, studies involving SARS-CoV proteins have demonstrated a possible role for IMPα/β1 in the viral infection. IMPα/β1 binds to the viral cargo protein in the cytoplasm and translocates it into the nucleus where the virus can compromise the host cell’s antiviral defense, and subsequently, increase the infection [185,186,187]. Along those lines, ivermectin was identified as a potent inhibitor of the replication of SARS-CoV-2 clinical isolates in vitro, with an IC*_50_* value of about 2.5 µM. The mechanism was hypothesized to be through the inhibition of importins, specifically IMPα/β1-facilitated nuclear import of viral proteins [188].

### 2.8. Niclosamide

Niclosamide (Niclocide) is a chlorinated salicylanilide derivative (Figure 8) and anthelminthic agent that is used for the treatment of tapeworm infections with a mechanism that involves uncoupling of oxidative phosphorylation. It also has antineoplastic activity that is attributed to its ability to induce androgen receptors degradation through a proteasome-mediated pathway [189,190]. The drug is being tried in patients to treat COVID-19 symptoms alone or in combination with diltiazem, which is also thought to have antiviral activity [191].

Niclosamide has been reported to have a broad antiviral activity against SARS-CoV, MERS-CoV, Zika virus, Ebola virus, flaviviruses, and adenoviruses, among others. Particularly, niclosamide inhibited SARS-CoV replication with an *EC_50_* value of <0.1 μM in VeroE6 cells, and significantly inhibited MERS-CoV replication at a concentration of 10 μM in VeroB4 cells [192,193]. Recently, it was shown that niclosamide exhibited very potent antiviral activity against SARS-CoV-2 (*IC_50_* = 0.28 µM) [194]. Multiple potential mechanisms have been reported to justify niclosamide’s antiviral activity, including the prevention of viral entry by altering endosomal pH and the prevention of viral replication by inhibiting autophagy [195].

### 2.9. Doxycycline

Doxycycline (Acticlate) is a semi-synthetic tetracycline antibiotic (Figure 8) with broad-spectrum anti-bacterial and anti-parasitic activities. It was first approved in the U.S. in 1967 and it is available in oral and parenteral dosage forms. It primarily inhibits bacterial protein synthesis by targeting the 30S subunit of the bacterial ribosomal RNA [196]. It is currently being tried in about five interventional clinical trials for COVID-19 patients. The drug is being evaluated alone and in combination with hydroxychloroquine or ivermectin.

Multiple reasons have been identified in the literature to support the use of doxycycline, and tetracyclines in general, to treat or prevent SARS-CoV-2 infection. Tetracyclines have broad-spectrum antimicrobial activity, significant distribution to the lung tissues, and substantial lipophilicity to penetrate the outer lipophilic layer of SARS-CoV-2. Furthermore, coronaviruses exploit the zinc-binding matrix metalloproteinases for cell infiltration and replication. Doxycycline can chelate with the zinc metal, and therefore, it may disrupt the activity of metalloproteinases and inhibit the viral entry, fusion, and/or replication [197,198,199]. As demonstrated with the dengue virus *in vitro*, doxycycline may also inhibit the replication of single-stranded RNA viruses via inhibiting the viral serine protease [200]. Moreover, tetracyclines are also known to promote anti-inflammatory activity by downregulating the NFκB pathway and by reducing the levels of pro-inflammatory cytokines such as IL-1β, IL-6, and TNF-α [201]. Certain tetracyclines can also induce activation of protein kinase C and apoptosis of mast cells, and thus, can decrease levels of circulating inflammatory agents [202,203,204]. Such activity is important in managing the cytokine storm triggered by SARS-CoV-2. Lastly, recent computational studies indicated that doxycycline can potentially inhibit PL^pro^ [95] and/or M^pro^ [205] of SARS-CoV-2.

### 2.10. Chlorpromazine

**Figure 9:**
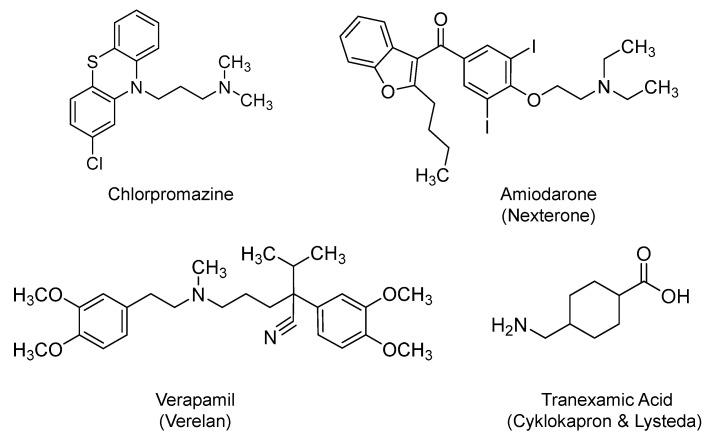
The chemical structures of alkylamine-based drugs that are currently being tested in COVID-19 patients. Presented are the antipsychotic phenothiazine chlorpromazine, the antiarrhythmic benzofuran-based amiodarone, the phenylalkylamine-based calcium channel blocker of verapamil, and the trans-stereoisomer of 4-(aminomethyl)cyclohexane-carboxylic acid antifibrinolytic agent, also known as tranexamic acid.

It is a phenothiazine derivative (Figure 9) that was first approved by the U.S. FDA in 1957. It is being prescribed as antipsychotic agent for the treatment of bipolar disorder, psychotic disorders, schizophrenia, among others. Its pharmacology is mainly attributed to blocking postsynaptic mesolimbic dopaminergic receptors in the brain. It has multiple other effects on other chemical neurotransmitters [206]. However, it has a U.S. black box warning because it is associated with an elevated mortality rate in elderly patients suffering from dementia-related psychosis [207]. Nevertheless, it is currently considered for evaluation in COVID-19 patients (NCT04366739 and NCT04354805).

One potential rationale behind these trials is that chlorpromazine is thought of as an inhibitor of clathrin-dependent endocytosis, a key mechanism for the viral entry into the host cells, as it was demonstrated in the case of mouse hepatitis virus [208], SARS-CoV, and MERS-CoV [36,209]. The basic chemical nature of chlorpromazine potentially modifies the acidic environment of lysosomes, key components of the endocytic pathway, and thus, chlorpromazine interferes with the early stage of the viral life cycle and prevents the subsequent viral genome release and replication. The potential benefit of chlorpromazine in COVID-19 patients has been brought to the front line as officials in GHU PARIS Psychiatrie and Neurosciences (Sainte-Anne hospital, Paris, France) reported a lower incidence of symptomatic and severe cases of COVID-19 infections among psychiatric patients (∼4%) relative to health care professionals (∼14%). The report argues that the biodistribution profile of chlorpromazine is particularly interesting as studies have reported a higher concentration of the drug in lungs (20–200-fold) and in saliva (30–100-fold) than in plasma, which is important given the respiratory nature of SARS-CoV-2 infection. Furthermore, the report claims that chlorpromazine may prevent the neurological forms of COVID-19 given its ability to reach the central nervous system [210]. In a recent study, chlorpromazine has been shown to exhibit in vitro antiviral activity against SARS-CoV at *IC_50_* < 10 µM (3.78-fold less than the *CC_50_*). The data indicated that the drug inhibits the entry stage of SARS-CoV infection by disrupting the virus’s spike protein-mediated fusion with the host cellular membrane. However, chlorpromazine did not block the viral replication in mouse lungs infected with SARS-CoV (MA15), yet it diminished weight loss and improved therapeutic outcomes, with the higher dose providing more protection [211].

### 2.11. Amiodarone and Verapamil

On one hand, amiodarone (Nexterone) is a benzofuran derivative (Figure 9) that is classified as a class III antiarrhythmic agent. Amiodarone was first approved by the U.S. FDA in 1985. It is orally or parenterally used to manage life-threatening recurrent ventricular fibrillation or recurrent ventricular tachycardia refractory to other antiarrhythmic agents or in patients intolerant of other agents used for these conditions [212]. It inhibits adrenergic stimulation and affects endogenous mono and di-cation channels. On the other hand, verapamil (Verelan) is a phenylalkylamine calcium channel blocker (Figure 9) that was first approved in the U.S. in 1981. It is orally or parenterally used in the treatment of hypertension, cardiac arrhythmia, and angina [213]. It is also classified as a class IV antiarrhythmic agent. The two drugs are being evaluated in COVID-19-hospitalized patients with symptoms (NCT04351763). Several studies have shown that amiodarone and verapamil can interfere with the viral entry and amplification by blocking calcium ion channels [214]. Amiodarone and verapamil may also accumulate into late endosomes/lysosomes, and given their chemical basicity, they may increase the pHs of these organelles. As a result, the two drugs may block the fusion of viral and endosomal membranes, prevent the release of the viral genetic material into the cytoplasm, and ultimately inhibit the virus replication [214,215,216,217,218].

Noteworthy is the fact that amiodarone has a U.S. boxed warning because it precipitates life-threatening arrhythmias, pulmonary toxicity, and hepatotoxicity. It is possible that its derivative, i.e., dronedarone, serves as a better alternative [212].

### 2.12. Tranexamic Acid

Tranexamic acid (Cyklokapron and Lysteda) is a synthetic analog of lysine (Figure 9). It promotes antifibrinolytic activity by reversibly binding to the lysine-binding sites on the kringle domains of plasminogen. It prevents the bioconversion of plasminogen to plasmin, a serine protease that breaks down the crosslinked fibrin in blood clots. Therefore, tranexamic acid is used to treat bleeding disorders as in heavy menstrual bleeding or during tooth extraction in patients with hemostatic defects. Tranexamic acid was first approved by the U.S. FDA in 1986 and is being prescribed orally and parenterally [219]. It is currently being evaluated as interventional therapy in COVID-19 infection at University of Alabama at Birmingham in randomized, placebo-controlled, double blind studies (NCT04338126 and NCT04338074). In the same vein, a proprietary formulation known as LB1148 comprised of tranexamic acid, poly-ethylene glycol, electrolytes, and sugar, with potential gastrointestinal protective activity, is also being evaluated for the treatment of pulmonary dysfunction associated with COVID-19 pneumonia (NCT04390217).

The rationale for the proposed testing of tranexamic acid stems from the fact that plasmin acts on SARS-COV-2 by cleaving a newly inserted furin site (RRAR/S) in the spike S protein portion of the virus. The plasmin-mediated cleavage is claimed to facilitate the viral entry and fusion, and therefore, its inhibitor may block the early stage of the viral life cycle. Furthermore, the detrimental effect of plasmin becomes more significant in SARS-COV-2 patients with diabetes, hypertension, coronary artery disease, cerebrovascular illness, kidney dysfunction, and lung disease because these patients commonly have elevated levels of plasmin(ogen). Thus, it was proposed that a better outcome can be achieved by blunting the catalytic activity of plasmin or its formation from plasminogen in infected patients having the above co-morbidities [41].

With respect to LB1148, the formulation is designed to neutralize the activity of potent digestive protease enzymes that can damage tissues and organs upon escaping the intestines through a compromised gastrointestinal mucosal barrier. Escaped digestive proteases trigger a life-threatening cascade of inflammation, cytokine storm, tissues autodigestion, and organ failure, including ARDS, all of which have been reported in severe and critically ill cases of COVID-19. Thus, LB1148 has been put forward for evaluation to reduce the above complications, given its ability to inhibit trypsin and trypsin-like proteases [220].

### 2.13. Spironolactone

**Figure 10:**
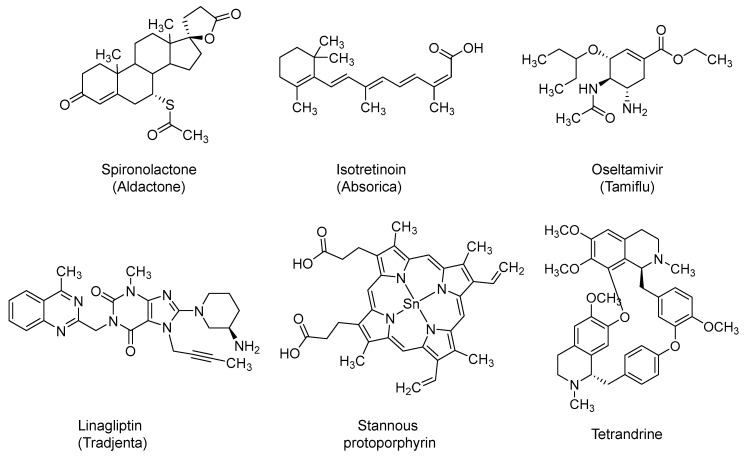
The chemical structures of miscellaneous agents that are also being tested in clinical trials for COVID-19 patients and exhibit inhibitory effects on the early events of the viral life cycle. Important among them are the steroid-based potassium sparing diuretic spironolactone, which has been shown to block the host androgen receptors, and thus, to decrease the transcription of TMPRSS2 gene. Linagliptin is dihydro-purinedione-based inhibitor of DPP-4, a functional receptor for the spike protein of MERS-CoV, and a potential receptor for SARS-CoV-2. It also exhibits therapeutically beneficial anti-inflammatory effects.

Spironolactone (Aldactone) is a steroid-based potassium sparing diuretic (Figure 10). It was first approved in the U.S. in 1960 to treat heart failure and hypertension [221]. It also has antiandrogenic effects [222]. It has also been used for treating acne in females as well as in male-to-female medical gender transition [222]. It is being evaluated as a possible treatment for COVID-19-induced ARDS alone (NCT04345887) or in combination with bromhexine (NCT04424134). Androgen receptor activity appears to be required for the transcription of TMPRSS2 gene. TMPRSS2 is the enzyme that activates the spike S protein of SARS-CoV-2 and subsequently facilitates the viral entry. By the virtue of blocking androgenic receptors, spironolactone may block the expression of TMPRSS2 and the viral entry [223].

Furthermore, it has been reported that spironolactone can increase ACE2 level in plasma by 3–5-fold [224]. The circulating form of ACE2 may inactivate SARS-CoV-2 by impeding its binding to the membrane bound ACE2 and the subsequent entry into pulmonary host cells. In fact, recombinant human soluble ACE2 has been put forward to protect against ARDS and death in COVID-19. Not only that, but the soluble ACE2 can also convert angiotensin II to angiotensin 1–7, and therefore, it can decrease the cardiovascular complications associated with the high level of angiotensin II in severe or critically ill cases of COVID-19 [225].

### 2.14. Isotretinoin

Isotretinoin (Absorica) (Figure 10) is a retinoid derivative of vitamin A. Isotretinoin, also known as 13-cis retinoic acid, was first approved by the U.S. FDA in 1982. It is used orally to treat severe recalcitrant nodular acne. The mechanism is not well understood, yet it may involve apoptosis induction in various cells, including sebaceous gland cells. Isotretinoin has a low affinity for retinoid receptors; however, its intracellular metabolites may act as better agonists for these receptors [226,227]. Few clinical trials appear to include the use of isotretinoin in COVID-19 patients. Isotretinoin has been identified as a strong down-regulator of ACE2; thus, it has been proposed to interfere with the viral entry [228]. Isotretinoin was also recently identified as a potential PL^pro^ inhibitor in a computational exercise [95]. Importantly, isotretinoin has been reported to significantly inhibit monocyte and neutrophil chemotaxis in acne patients indicating potential anti-inflammatory effects [229].

### 2.15. Oseltamivir

Oseltamivir (Tamiflu) is *N*-acetyl sialic acid (*N*-acetylneuraminic acid) analog (Figure 10) and active site inhibitor of neuraminidase. It was first approved in 1999 in the U.S. as an orally administered prophylaxis or treatment for seasonal influenza [230,231]. It has also been used for H7N9 and H5N1 flu viruses [232,233]. It is currently being tested alone or in combination with hydroxychloroquine, azithromycin, lopinavir/ritonavir, or ASC09F for the prevention and/or treatment of SARS-CoV-2 infection. Mechanistically, oseltamivir is a prodrug that is hydrolyzed to the active carboxylate form. The carboxylate form inhibits the influenza virus spread by inhibiting neuraminidase, an enzyme that cleaves the glycosidic bond between hemagglutinin of the new virus and sialic acid of the host cell. This leads to viral aggregation with no further infectivity. Oseltamivir is likely to prevent/limit the flu infection during the COVID-19 pandemic, rather than being a treatment for SARS-CoV-2 itself [14]. In fact, neither oseltamivir nor zanamivir has demonstrated activity against SARS-CoV in in vitro cell cultures [234].

### 2.16. Dipeptidyl Peptidase-4 (DPP-4) Inhibitors: Linagliptin and Sitagliptin

Linagliptin (Tradjenta) (Figure 10) is a potent, orally bioavailable dihydro-purinedione-based inhibitor of DPP-4. Inhibiting DDP-4 results in an increased level of incretins, which subsequently regulate glucose homeostasis by increasing the pancreatic release of insulin and decrease the pancreatic release of glucagon. The drug was first approved by the U.S. FDA in 2011 as an oral antidiabetic agent for the treatment of type II diabetes mellitus [235]. It is being evaluated in the context of COVID-19 pandemic in two trials (NCT04341935 and NCT04371978). Likewise, sitagliptin (Januvia; 2006), another DPP-4 inhibitor, is also being tested in COVID-19-positive diabetic patients (NCT04365517). Yet, the use of DPP-4 inhibitors in the treatment of COVID-19 patients requires extensive investigation. Reports that support the potential benefits of DPP-4 inhibitors in COVID-19 patients mention that DPP-4 was identified as a functional receptor for the spike protein of MERS coronavirus [236] and that antibodies targeting DPP-4 protein inhibited human coronavirus-Erasmus Medical Center infection of Huh-7 cells and primary human bronchial epithelial cells [34]. Therefore, DPP-4 inhibitors have been proposed to prevent the entry of SARS-CoV-2 into the human cells, offering a significant prophylactic/treatment opportunity. Furthermore, anti-inflammatory effects, including decreased production of pro-inflammatory cytokines and reduced macrophage infiltration, have also been reported with the use of DPP-4 inhibitors [237]. The anti-inflammatory effects of DPP-4 inhibitors have been projected to mitigate the excessive inflammation reported in severe and critically ill cases of COVID-19 [237].

### 2.17. Stannous Protoporphyrin (also Reported as RBT-9)

The molecule is a cyclic tetra-pyrrole derivative (Figure 10) that is widely described as heme oxygenase-1 inhibitor. It is being tested in COVID-19 patients in two clinical trials (NCT04364763 and NCT04371822). Its role in treating COVID-19 patients is not well understood, yet it could be because of its antiviral activity and/or immunomodulatory effects. On one hand, stannous protoporphyrin exhibited in vitro antiviral activity against yellow fever virus and dengue virus [238] and against Zika virus, Chikungunya virus, and other arboviruses, potentially by targeting the viral envelope [239]. Furthermore, the molecule also appears to modulate inflammatory responses in multiple animal models including those of osteoarthritis [240,241,242].

### 2.18. Tetrandrine

Tetrandrine is a bis-benzylisoquinoline alkaloid (Figure 10) that is isolated from the plant *Stephania tetrandra*, and other Asian herbs [243]. It has a host of biological activities, including antiallergic, antiarrhythmic, and vasodilatory effects [244]. Tetrandrine also has antiviral and anti-inflammatory/immunomodulatory activities which represent the foundation for the ongoing clinical trial in COVID-19 patients (NCT04308317). Along these lines, tetrandrine potently inhibited herpes simplex virus type-1-induced keratitis in BALB/c mice [245]. Furthermore, tetrandrine also inhibited human coronavirus OC43 infection of MRC-5 human lung cells by inhibiting the expression of HCoV-OC43 spike and nucleocapsid proteins [246]. Tetrandrine also inhibited the entry of Ebola virus into host cells in vitro and demonstrated some efficacy against Ebola in mouse models [247]. It was also reported that tetrandrine could potently inhibit pro-inflammatory responses in lipopolysaccharide-treated mice [248].

### 2.19. Janus Kinases (JAKs) Inhibitors: Baricitinib, Ruxolitinib, and Tofacitinib

**Figure 11:**
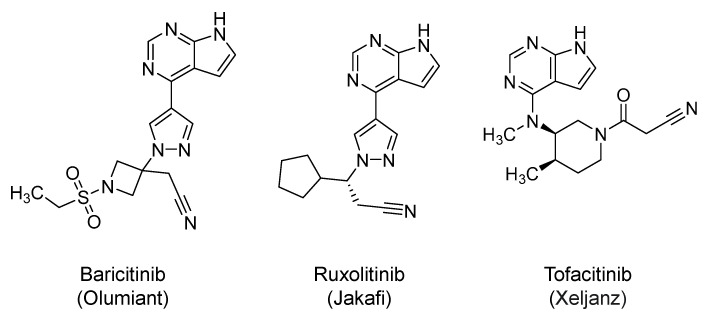
The chemical structures of pyrrolo-pyrimidine-based JAK inhibitors. The drugs potentially block clathrin-mediated endocytosis of the virus. They also exhibit anti-inflammatory/immune-modulatory agents.

Baricitinib (Olumiant; 2018) (Figure 11) is a 4-substituted, pyrrolo-pyrimidine-based inhibitor of JAK1/2. The drug is orally approved for the treatment of rheumatoid arthritis [249]. Currently, it is being evaluated in several clinical trials to determine its safety and efficacy in COVID-19 patients. Baricitinib is included in the next iteration of the U.S. National Institute of Allergy and Infectious Diseases Adaptive COVID-19 Treatment Trial 2 (ACTT 2) (NCT04401579) in which remdesivir is being evaluated with and without baricitinib in COVID-19 patients. Baricitinib has been predicted to decrease the virus ability to infect lung cells and to disrupt the intracellular assembly of virus particles by binding to AP_2_-associated protein kinase 1, a regulator of endocytosis. It has also been shown that the drug binds to and disrupts the cyclin G-associated kinase, another endocytosis regulator [250]. Thus, JAKs inhibitors including baricitinib have been put forward as potential inhibitors of the viral entry.

Furthermore, it is well known that SARS-CoV-2 infection is associated with an excessive proinflammatory immune response, which is potentially related to overactivated JAKs. The cytokines involved in this response are IL-1, IL-2, IL-4, IL-6, IL-10, IL-12, IL-13, IL-17, IP-10, IFN-γ, GCSF, HGF, MCSF, MCP-1, MIP-1α, and TNF-α. The excessive release of cytokines results in significant organ damage and increased an mortality rate among the severely ill patients [18,251]. In this arena, JAKs phosphorylate the cytokine receptors, which in turn, recruit signal transducers and activators of transcription (STATs) to eventually modulate gene expression. The JAK-STAT signaling has been found to play a significant role in signal transduction in hematopoietic cells and immune system cells. In fact, JAKs inhibitors have been shown to exert potent anti-inflammatory and immunosuppressive effects by targeting components of the innate and adaptive immune system, including dendritic cells, natural killer cells, and regulatory T and T helper cells [252,253,254]. Together, baricitinib and other JAKs inhibitors are expected to be beneficial in COVID-19 patients because they inhibit JAK1 and JAK2- mediated proinflammatory cytokine release. Importantly, baricitinib has minimal interactions with CYP450 enzymes and drug transporters, which permits its use in combination with different antiviral agents. Along these lines, the use of baricitinib (4 mg/day) in combination with lopinavir/ritonavir was evaluated in COVID-19 patients (N = 12) with moderate pneumonia in a small, open-label study in Italy. Baricitinib was well tolerated with no serious adverse events. Within two weeks, baricitinib-treated patients had substantial improvement in pulmonary function parameters and required no admission to ICU [255].

Likewise, ruxolitinib (Jakafi; 2011) (Figure 11) is orally approved for the treatment of graft-versus-host disease, myelofibrosis, and polycythemia vera [256,257,258]. Structurally, it is 4-substituted pyrrolo-pyrimidine derivative. Mechanistically, it is a kinase inhibitor which selectively inhibits JAK1 and JAK2 (*IC_50_* = 2.8–3.3 nM). Given its structural and mechanistic similarity to baricitinib, ruxolitinib is also expected to promote similar antiviral and anti-inflammatory/immunomodulatory effects in COVID-19 patients [252,253,254]. Accordingly, about 14 interventional clinicals trials are now evaluating the potential safety and efficacy of ruxolitinib in patients with COVID-19. The trials are testing ruxolitinib alone or in combination with anakinra (NCT04366232), tocilizumab (NCT04424056), or simvastatin (NCT04348695). Lastly, tofacitinib (Xeljanz; 2012) [259], a third JAKs inhibitor, is also under consideration to protect against lung injury in COVID-19 patients.

## 3. Potential Macromolecular Inhibitors of Early Viral Events in Clinical Trials

### 3.1. Heparins

**Figure 12:**
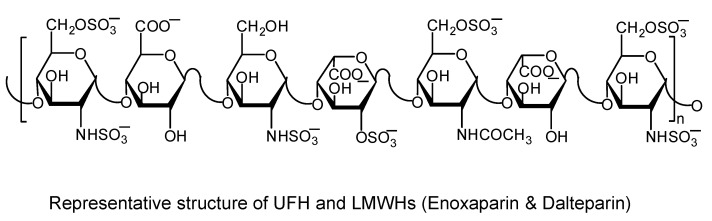
A representative chemical structure of UFH and LMWHs. These sulfated glycosaminoglycans exhibit remarkable antithrombin-mediated anticoagulant activity and anti-inflammatory and antiviral effects.

Unfractionated heparin (UFH) is a heterogeneous mixture of sulfated polysaccharides (Figure 12) (also known as sulfated glycosaminoglycans) with an average molecular weight of 15 kDa. Chemical and enzymatic depolymerizations of UFH have led to low molecular weight heparins (LMWHs) including enoxaparin and dalteparin with an average molecular weight of 5 kD. Other LMWHs include tinzaparin and bemiparin. Heparins are anticoagulants that promote effects via activating antithrombin, which in turn leads to the inhibition of thrombin and factor Xa, two procoagulants in the common coagulation pathway [260].

Heparan sulfate proteoglycans, proteins that are heavily decorated with sulfated glycosaminoglycans, have been reported to act as cellular receptors for several viruses including herpes simplex viruses, HIV, dengue virus, and many others [261]. Earlier, it was also shown that binding of human CoV-NL63 to heparan sulfates was required for viral attachment and infection of target cells [262]. Interestingly, it was also shown that treating HEK293E/ACE2-Myc cells with heparinase or exogenous heparins prevents the binding of the viral spike protein to the host cells. Such treatment also inhibited SARS pseudovirus infection, indicating that heparan sulfate proteoglycans may also provide binding sites for SARS-CoV attachment at the early phase of invasion. The results reveal that, in addition to ACE2, heparan sulfate proteoglycans are vital cell-surface molecules for SARS-CoV cell entry [263]. Very recently, heparins were reported to bind to the receptor–binding domain of COVID-19 spike S protein, leading to a conformational change that blocks the viral attachment and/or entry, and subsequently, the cellular invasion by SARS-CoV-2 [264,265].

Besides its antiviral activity, the anticoagulant activity of heparins is also very important. Severely affected COVID-19 patients are reported to have a hypercoagulable state, which has been linked to poor clinical outcomes including progressive respiratory failure, ARDS, and death [24,27,266]. Macrovascular and diffuse microvascular thrombi in the lungs, heart, liver, and kidneys have been reported in critically ill patients [267]. In fact, elevated levels of D-dimer, fibrinogen, and prothrombotic cytokines have also been reported in COVID-19 patients. In particular, patients with an elevated D-dimer level were more likely to die in hospital [268,269,270]. Moreover, IL-6 and TNF-α levels have also been reported to be significantly higher in ICU-patients which also put them at a greater risk of developing venous thromboembolism complications [270,271]. Early anticoagulation in patients with severe COVID-19 infection has been shown to reduce the risk of thrombotic complications and improve clinical outcomes [266,267]. Additional therapeutic benefits of heparins are promoted by their anti-inflammatory effects. Heparins have been reported to promote anti-inflammatory effects against sepsis and ARDS by neutralizing the activity of pro-inflammatory proteins such as histones and high-mobility group box 1 [272,273]. The potential of LMWHs to mitigate cytokine storm in severe COVID-19 patients has recently been described in a retrospective clinical study [274]. Given the antiviral, anticoagulant, and anti-inflammatory effects, more than 20 interventional clinical trials are now evaluating the benefits of heparins in COVID-19 patients.

### 3.2. DAS181

DAS181 (Fludase) is a recombinant sialidase enzyme that cleaves sialic acid on the surface of pulmonary epithelial cells [275,276,277]. Several viruses are reported to use sialic acid as a receptor while infecting the epithelial cells. Thus, treatment with DAS181 blocks the viral entry and prevents the viral infection. Interestingly, DAS181 is delivered using a nebulized formulation which is suitable for critically ill patients. The enzyme has demonstrated a broad antiviral activity spectrum against several sialic acid-dependent viruses, including respiratory viruses [278,279,280]. The drug is currently being evaluated in a phase IIb clinical trial in China for the treatment of hospitalized patients with severe influenza infections (STOP FLU). The drug is also being evaluated in a multicenter, worldwide phase III clinical trial for the treatment of immunocompromised, hospitalized patients with lower respiratory tract parainfluenza virus infections (STOP PIV) (NCT03808922). The above trials and others (NCT04324489 and NCT04354389) also include COVID-19 patients.

### 3.3. Recombinant Human ACE2 (rhACE2; also Reported as APN01)

Because the host ACE2 protein has been identified as a main receptor for SARS-CoV-2, its soluble recombinant human form i.e., rhACE2 has been put forward as a parenterally administered inhibitor of SARS-CoV-2 entry into the host cells. In fact, the recombinant human receptor was shown to inhibit SARS-CoV-2 infection of Vero-E6 cells in a dose-dependent fashion. Likewise, it inhibited the SARS-CoV-2 infection of human capillary organoids and that of human kidney organoids [281]. Accordingly, the rhACE2 can eventually block the virus replication and spread, and subsequently, can prevent the anticipated lung injury. The rhACE2 was also shown to generally decrease the concentration of IL-6, a proinflammatory cytokine reported to increase in severe COVID-19 cases [282]. Therefore, rhACE2 also has the potential to mitigate COVID-19-associated excessive inflammation. Importantly, rhACE2 can increase the conversion of angiotensin II to angiotensin 1-7, and thus, it decreases the cardiovascular complications that are expected to arise because of the high level of angiotensin II in severe or critically ill cases of COVID-19 [225]. Currently, rhACE2 (APN01) is being evaluated in phase 2 clinical trial (NCT04335136).

### 3.4. Combination of REGN10933 and REGN10987

This is a cocktail of two noncompeting monoclonal antibodies, each of which identifies distinct binding spot on the receptor binding domain of SARS-CoV-2 spike S protein. The cocktail is expected to block the viral entry and its spread. The two antibodies were identified following a series of experiments using genetically humanized VI mice and B cells derived from convalescent patients [283]. REGN10933 was found to bind at the top of the receptor binding domain of the spike S protein, extensively overlapping with the binding site for ACE2. The epitope for REGN10987 is located on the side of the receptor binding domain, away from the REGN10933 epitope, with no overlap with the ACE2 binding site. The neutralization potencies of the two monoclonal antibodies are within the picomolar range [283]. Importantly, the cocktail concept is being considered to overcome potential issues that may arise because of the virus mutations. The antibody cocktail is now being tested in human trials (NCT04426695 and NCT04425629).

### 3.5. COVID-19 Convalescent Plasma and Immunoglobulins

Human plasma from patients who have recently recovered from COVID-19 is known as COVID-19 convalescent plasma. This plasma contains antibodies against SARS-CoV-2, and therefore, it has been used to provide passive immunity against the virus which may help prevent the infection and/or accelerate the patients recovery from the infection [284,285,286,287]. The mechanisms by which convalescent plasma helps COVID-19 patients include neutralizing antibodies (IgG and IgM) that are mainly directed towards the spike S protein and other plasma components that mitigate the excessive inflammation and the hypercoagulable state [288]. Importantly, the strategy was previously used in the treatment of other viral infections with variable levels of success. In SARS-CoV patients, the use of convalescent plasma was reported to reduce the hospitalization duration and to decrease the mortality rate, particularly if the treatment was initiated in the early stage of the infection [289,290,291,292]. With respect to SARS-CoV-2, there are few small trials reporting on the use of COVID-19 convalescent plasma.

In uncontrolled pilot study in China, adults (N = 10) with severe forms of COVID-19 were treated with a single convalescent plasma transfusion along with the standard care. The median time from the symptoms onset to the transfusion was about 16.5 days. After the transfusion, the virus RNA was undetectable in all patients within 6 days. The symptoms were reported to improve in all patients within 24–72 h with the patients also showing substantial radiologic pulmonary recovery [293]. Improvements were also reported by uncontrolled case series in China in critically ill adults (N = 5) who had high viral loads regardless of the antiviral treatment. The patients appeared to suffer from rapidly progressing severe COVID-19 and ARDS requiring mechanical ventilation. The patients were treated with two transfusions of convalescent plasma (10–22 days after hospitalization) and continued to receive antiviral treatments and methylprednisolone. After the transfusions, the fever was resolved within 3 days in most patients, titers of neutralizing antibody increased in all patients, organ failure assessment scores improved in all patients, and viral loads substantially decreased within 12 days [294]. Similar improved clinical outcomes were reported by uncontrolled case series in the U.S. in adults (N = 25) having severe and/or life-threatening COVID-19 [295] and by uncontrolled descriptive study in China (N = 6) [296]. Nevertheless, an open-label, randomized, controlled study in China in adults (N = 103) with severe and/or life-threatening COVID-19 reported no statistically significant difference between convalescent plasma-treated patients and those who were treated by the standard of care, with respect to time to clinical improvement within 28 days, time to hospital discharge, or mortality [297]. In fact, a recent systematic review evaluated published trials for convalescent plasma in adults with COVID-19 and indicated limited efficacy and potential safety problems pertaining to the use of convalescent plasma [298]. Together, the safety and efficacy of COVID-19 convalescent plasma for the treatment of COVID-19 is yet to be rigorously established in large trials.

Likewise, the use of commercially available or investigational SARS-CoV-2 IV-administered immunoglobulins (IGIV, IVIG, Octagam, Flebogamma DIF, and γ-globulin) have been reported in COVID-19 patients. They are generally used as a replacement therapy to provide passive immunity against immunodeficiency diseases including viral infections, and in this case against SARS-CoV-2 [299,300,301]. The benefit of these immunoglobulins in the treatment of COVID-19 continues to evolve. For example, COVID-19 case reports from China indicated that treatment with the immunoglobulins IGIV at the early stage provided some clinical benefits in adults (N = 3) with severe COVID-19 [302]. Furthermore, a retrospective study from China also revealed that treating patients (N = 58) of severe or critical COVID-19 illness in ICU with IGIV (in addition to antiviral and anti-inflammatory agents) within 48 h of admission resulted in decreased hospitalization duration, reduced length of ICU time, and reduced need for mechanical ventilation [303].

Overall, more than 100 clinical trials continue to assess the use of convalescent plasma and/or immunoglobulins so as to evaluate their efficacy and safety.

## 4. Conclusions

The life cycle of SARS-CoV-2 can be generally classified into two stages: the initial/early stage and the advanced/late stage. The initial/early stage includes events prior to the viral RNA replication and involves the virus attachment/adsorption, fusion, and endocytosis, whereas the advanced/late stage involves the RNA replication, viral protein synthesis and processing, and viral particle assembly and release. Several viral and host proteins are important for each stage. These proteins and the corresponding events serve as potential drug targets for the design and development of anti-COVID-19 therapeutics. In this review we summarized potential therapeutics that interfere with the early events of the viral cycle. Important drug targets in this stage are spike S protein, ACE2, TMPRSS2, and clathrin-mediated endocytosis.

In this paper, we described potential therapeutics that are currently listed in clinicaltrials.gov. On the one hand, these include small molecule drugs such as quinoline-based antimalarials ((hydroxy)-chloroquine and others), RAAS modifiers (captopril, losartan, and others), statins (atorvastatin and simvastatin), guanidino-based serine protease inhibitors (camostat and nafamostat), antibacterials (macrolides, clindamycin, and doxycycline), antiparasitics (ivermectin and niclosamide), cardiovascular drugs (amiodarone, verapamil, and tranexamic acid), antipsychotics (chlorpromazine), antivirals (umifenovir and oseltamivir), DPP-4 inhibitors (linagliptin), JAK inhibitors (baricitinib and others), and few others. On the other hand, they also include macromolecules, including sulfated glycosaminoglycans (UFH and LMWHs) and polypeptides such as the enzymes DAS181 and rhACE2. They also include the viral spike protein-targeting monoclonal antibodies REGN10933 and REGN10987. Many of the above drugs are currently approved therapeutics for other indications, and thus, they present a unique repurposing opportunity. Yet, others are new molecular entities such as DAS181, rhACE2, REGN10933, and REGN10987. Interestingly, the presented potential therapeutics exploit a wide spectrum of mechanistic strategies which will necessarily enhance the likelihood of obtaining effective therapeutics in a timely fashion. Importantly, many of the above drugs promote antiviral effects by other mechanisms beyond the inhibition of the viral entry. One interesting example is umifenovir.

Furthermore, many of the presented therapeutics exert pharmacological effects beyond antiviral activity. Of note are the cardiovascular protective effects of RAAS modifiers, statins, and DPP-4 inhibitors. Likewise, the anti-inflammatory/immunomodulatory effects of (hydroxy)chloroquine, statins, DPP-4 inhibitors, macrolides, nafamostat, clindamycin, doxycycline, JAK inhibitors, rhACE2, and heparins are of enormous significance given the reported excessive inflammation in the severe cases of COVID-19. The anticoagulant effects of heparins, camostat, and nafamostat also offer lifesaving therapeutic benefits by preventing and/or treating the virus-triggered coagulopathies. In fact, considering the multifaceted clinical presentations of COVID-19, the antiviral, anti-inflammatory, and anticoagulant activities of heparins are remarkably unique.

Lastly, the individual use of many of the described therapeutics may eventually become beneficial as a prophylaxis and/or as a treatment for the mild cases of the disease. Nevertheless, it is important to emphasize here that a combination of the above drugs with each other and/or with those that impact the advanced/late stage of the viral life cycle will likely have a better chance to succeed in combating the pandemic and treating the critically ill patients. The combinations will likely prevent the deadly progress of the diseases, alleviate symptoms faster, and avoid loss of therapeutic efficacy because of potentially significant viral mutations. Based on initial results, statins, nafamostat, azithromycin, JAK inhibitors, heparins, rhACE2, DAS181, and the spike protein-targeting monoclonal antibodies (REGN10933 and REGN10987) appear to carry the most promising therapeutic effects.

## Figures and Tables

**Table 1:** The potential anti-COVID-19 therapeutics that are being tested in clinical trials based on targeting the early events of the life cycle of SARS-CoV-2 ^(a)^.

**Small Molecules**
Quinoline-based drugs: (Hydroxy)chloroquine, mefloquine, & primaquine
Renin-angiotensin-aldosterone system (RAAS) modifiers: Captopril, losartan, & others
Hydroxy methylglutaryl CoA (HMG CoA) reductase inhibitors: Atorvastatin & simvastatin
Transmembrane protease serine 2 (TMPRSS2) inhibitors: Camostat, nafamostat, & others
Umifenovir
Macrolides: Azithromycin, clarithromycin, & carrimycin
Ivermectin
Niclosamide
Doxycycline
Chlorpromazine
Amiodarone and verapamil
Tranexamic acid
Spironolactone
Isotretinoin
Oseltamivir
Dipeptidyl peptidase-4 (DPP-4) inhibitors: Linagliptin and sitagliptin
Stannous protoporphyrin
Tetrandrine
Janus kinases (JAKs) inhibitors: Baricitinib, ruxolitinib, & tofacitinib
**Macromolecules**
Unfractionated heparin (UFH) and low molecular weight heparins (LMWHs)
DAS181
Recombinant human ACE2 (rhACE2)
REGN10933 and REGN10987
COVID-19 convalescent plasma and immunoglobulins

^(a)^ Some of the listed therapeutics have multiple antiviral mechanisms. Some also have anti-inflammatory and/or anticoagulant effects.

## Abbreviations

ACE2	Angiotensin converting enzyme-2
ARDS	Acute respiratory distress syndrome
3CL^pro^	3-Chymotrypsin-like protease
CC_50_	Half maximal cytotoxic concentration
COVID-19	Coronavirus disease-2019
DPP-4	Dipeptidyl peptidase-4
EC_50_	Half maximal effective concentration
HIV	Human immunodeficiency virus
HMG CoA	Hydroxymethyl glutaryl coenzyme A
*IC_50_*	Half maximal inhibitory concentration
ICU	Intensive care unit
LMWHs	Low molecular weight heparins
MERS	Middle East respiratory syndrome
M^pro^	Main protease
NSPs	Nonstructural proteins
PL^pro^	Papain-like protease
RAAS	Renin-angiotensin-aldosterone system
RdRp	RNA-dependent RNA polymerase
rhACE2	Recombinant human ACE2
SARS-CoV-2	Severe acute respiratory syndrome-coronavirus-2
TMPRSS2	Transmembrane protease serine 2
UFH	Unfractionated heparin
